# Smoking, alcohol, and diet in relation to risk of pancreatic cancer in China: a prospective study of 0.5 million people

**DOI:** 10.1002/cam4.1261

**Published:** 2017-12-22

**Authors:** Yuanjie Pang, Michael V. Holmes, Yu Guo, Ling Yang, Zheng Bian, Yiping Chen, Andri Iona, Iona Y. Millwood, Fiona Bragg, Junshi Chen, Liming Li, Christiana Kartsonaki, Zhengming Chen

**Affiliations:** ^1^ Clinical Trial Service Unit & Epidemiological Studies Unit (CTSU) Nuffield Department of Population Health University of Oxford Oxford United Kingdom; ^2^ Medical Research Council Population Health Research Unit (MRC PHRU) Nuffield Department of Population Health University of Oxford Oxford United Kingdom; ^3^ National Institute for Health Research Oxford Biomedical Research Centre Oxford University Hospital Old Road Oxford OX3 7LE UK; ^4^ Chinese Academy of Medical Sciences 9 Dongdan San Tiao Beijing 100730 China; ^5^ National Center for Food Safety Risk Assessment 37 Guangqu Road Beijing 100021 China; ^6^ School of Public Health Peking University Beijing 100191 China

**Keywords:** Alcohol, Chinese, fresh fruit, meat, pancreatic cancer, smoking

## Abstract

In China, the incidence of pancreatic cancer (PC) has increased in recent decades. However, little is known about the relevance to PC risk of lifestyle and behavioral factors such as smoking, alcohol drinking, and diet. The China Kadoorie Biobank prospective study recruited 512,891 adults (210,222 men, 302,669 women) aged 30–79 (mean 52) years from 10 diverse areas during 2004–08. During ~9 years of follow‐up, 688 incident cases of PC were recorded among those who had no prior history of cancer at baseline. Cox regression yielded adjusted hazard ratios (HR) for PC associated with smoking, alcohol and selected dietary factors. Overall, 74% of men were ever‐regular smokers and 33% of men drank at least weekly, compared with only 3% and 2% of women, respectively. Among men, current regular smoking was associated with an adjusted HR of 1.25 (95% CI 1.08–1.44) for PC, with greater excess risk in urban than rural areas (1.46 [1.19–1.79] vs 1.04 [0.86–1.26]). Heavy, but not light to moderate, alcohol drinking (i.e. ≥420 g/week) was associated with significant excess risk (1.69 [1.21–2.37]), again more extreme in urban than rural areas (1.93 [1.29–2.87] vs 1.35 [0.74–2.48]). Overall, regular consumption of certain foodstuffs was associated with PC risk, with adjusted daily vs never/rare consumption HRs of 0.66 (0.56–0.79) for fresh fruit and 1.16 (1.01–1.33) for red meat. In China, smoking and heavy alcohol drinking were independent risk factors for PC in men. Lower fresh fruit and higher red meat consumption were also associated with higher risk of PC.

## Introduction

Pancreatic cancer (PC) ranks as the sixth leading cause of cancer death globally 1 and its incidence and mortality have increased steadily in recent decades in China 2. Among all cancers, PC has the highest case fatality rate, with a 5‐year survival of <5% 1, 3. Although its pathogenesis remains poorly understood, PC has a multifactorial aetiology, with several known risk factors such as age, male sex, smoking, heavy alcohol drinking, obesity, diabetes, history of chronic pancreatitis, and family history of PC 4. Moreover, certain dietary factors have been implicated as risk factors for PC, including lower intake of fruit and vegetable and higher intake of red meat 4.

Of the known modifiable risk factors for PC, tobacco smoking is the most well‐established, with an attributable fraction of 25% in typical Western populations where widespread cigarette smoking among young adults has persisted for several decades 4. Compared with never smokers, current smokers typically carry a 50–70% excess risk 5. Heavy alcohol consumption (i.e. ≥3 drinks per day) is also associated with a 30% higher risk, but the effects of light to moderate alcohol drinking remain unclear 6. A few studies have also suggested that the association may vary by types of alcohol, with the excess risk greater for spirits than for other types of alcohol, such as beer or wine 7, 8, 9. Furthermore, prospective studies have been inconclusive on whether fresh fruit, vegetables, and red meat are associated with risk of PC 10, 11, 12. Previous prospective studies from East Asia have reported increased risks of PC among current and former smokers and a null association with alcohol drinking 5, 6, while for dietary factors the prospective evidence is very limited.

Most of the previous evidence is based on studies in Western populations, with little prospective data from low‐ and middle‐income countries, especially China, where lifestyle differs appreciably from high‐income countries. In China, for example, despite the rapid increase in per capita cigarette and alcohol consumption, smoking and drinking remain predominately a male phenomenon, and few adult women smoke cigarettes persistently from early adulthood, especially among those who were born after 1950 13. Similarly, although regular and episodic heavy drinking are now relatively common in Chinese men 14, this represents only a recent phenomenon, following rapid economic development, and chiefly involves strong spirits. In addition, consumption of fresh vegetables is high, whereas consumption of fresh fruit, red meat, and processed meat is low 15. Appropriate understanding of modifiable risk factors for PC in different populations is therefore needed to inform prevention strategies for this highly fatal disease globally. Our previous analyses have suggested that diabetes and obesity are metabolic risk factors for PC in the Chinese population 16, 17, as shown in other East Asian populations 4. We examined the prospective associations of smoking, alcohol, and certain dietary factors with incidence of PC in the China Kadoorie Biobank (CKB) study.

## Methods

### Study population

Details of the CKB design, survey methods, and population characteristics have been described elsewhere 18. Briefly, 512,891 participants (210,222 men and 302,669 women) aged 30–79 years were recruited into the study from 10 geographically defined localities (5 urban and 5 rural) in China during 2004–2008. The study areas were selected to provide diversity in risk exposure and disease patterns, while taking into account population stability, quality of mortality and morbidity registries, capacity, and long‐term commitment within the areas. Prior international, national, and regional ethical approvals were obtained, and all participants provided written informed consent.

### Data collection

At local study assessment clinics, participants completed an interviewer‐administered laptop‐based questionnaire on sociodemographic characteristics, smoking, alcohol consumption, diet, tea drinking, physical activity, personal and family medical history, and current medication. Personal medical history included history of diabetes, coronary heart disease (CHD), stroke, cancer, and other major conditions. Family medical history included diabetes, CHD, stroke, psychiatric disorders, and cancer in parents, siblings, or children. A range of physical measurements were recorded by trained technicians, including height, weight, hip and waist circumference, bioimpedance, lung function, blood pressure, and heart rate, using calibrated instruments with standard protocols.

Smoking status was classified as never (smoked <100 cigarettes in lifetime), occasional, former, or regular smoker 13. Former smokers were defined as those who had smoked a total of at least 100 cigarettes or equivalent but had quitted smoking for ≥6 months. Regular smokers were defined as those who reported having ever smoked one or more cigarettes (or their equivalent) daily for at least 6 months. Among regular smokers who had stopped 6 or more months before recruitment, approximately half did so because of ill health, and they were still counted as smokers in the main analyses. Among regular smokers, the amount of smoking was categorized as <20, 20–25, and ≥25 cigarettes per day.

Assessment of alcohol consumption has been described elsewhere 14. Briefly, data collected on alcohol drinking included whether the participant had drunk alcohol regularly (i.e. at least once a week on a regular basis) during the past 12 months, and, if so, the age at which drinking began, the type (beer, wine, or spirits), and the amount of each type consumed on a typical drinking week. The total amount consumed was calculated as grams (g) of pure alcohol, based on the beverage type and amount drunk, assuming the following alcohol content by volume (v/v): beer 4%, grape wine 12%, rice wine 15%, weak spirits 38%, and strong spirits 53%. Only one beverage type was allowed to be reported for a typical drinking day, and this defined the type of beverage drunk habitually. Weekly intake categories included abstainers, and among weekly drinkers, <140, 140–280, 280–420, and ≥420 g of alcohol per week. Abstainers were defined as those who had never or almost never drank alcohol in the past 12 months and had not drunk weekly in the past. Occasional drinkers were defined as those who in the past 12 months had drunk alcohol occasionally, during certain seasons, or monthly but less than weekly, and had not drunk weekly in the past. Reduced‐intake drinkers were those who in the past 12 months had drunk alcohol occasionally, during certain seasons, or monthly but less than weekly, but had drunk weekly in the past. Ex‐weekly drinkers were those who had drunk weekly in the past but had never or almost never drank alcohol in the past 12 months. Weekly drinkers were those who usually drank at least once a week during the past 12 months. Heavy drinking episodes were defined as the consumption of ≥60 g of alcohol on one occasion for men, and ≥40 g for women on a weekly basis.

Dietary data covered 12 major food groups: rice, wheat products, other staple foods, red meat, poultry, fish, eggs, dairy products, fresh vegetables, preserved vegetables, fresh fruit, and soybean products. Respondents were asked about the frequency of habitual consumption during the previous 12 months and chose among five categories of frequency (daily, 4–6 days per week, 1–3 days per week, monthly, or never or rarely). In this study, we focused on six food groups a priori that have been hypothesized to be associated with risk of PC, including fresh fruit, soybean products, red meat, fish, poultry, and dairy products 4.

### Resurveys

After completion of the baseline survey, 5–6% of the original participants were randomly selected for two resurveys, using similar procedures and questionnaires to those at the baseline. The first resurvey was conducted from July to October 2008 (*n* = 19,788), and the second resurvey from August 2013 to September 2014 (*n* = 25,242).

### Mortality and morbidity follow‐up

The vital status of each participant was determined periodically through China CDC's Disease Surveillance Points (DSP) system 19, supplemented by regular checks against local residential records and health insurance records and by annual active confirmation through street committees or village administrators. In addition, information about occurrence of major diseases and any episodes of hospitalisation was collected through linkage, via each participant's unique national identification number, with disease registries (for cancer, ischemic heart disease, stroke, and diabetes) and national health insurance claims databases. All events were coded using International Classification of Diseases 10th Revision (ICD‐10) by trained staff who were blinded to baseline information 18. By 1.1.2016, 37,290 (7%) participants had died, 3,898 (0.8%) were lost to follow‐up and 24,414 (4.8%) had developed cancer, including 702 (0.14%) with PC (ICD‐10 C25).

### Statistical analyses

The present study excluded 2577 participants with a prior history of cancer at baseline, leaving 510,314 participants and 688 PC cases for the main analysis. Very few women in the study smoked or drank alcohol, so the main analyses for smoking and alcohol were restricted to male participants (*n* = 209,290).

The prevalence and mean values of participants’ baseline characteristics by pancreatic cancer status were calculated using direct standardisation to the age (in 5‐year age groups), sex, and area structure of the CKB population. The calculations were done separately in PC cases and participants without PC applying the age, sex, and area distribution of the entire CKB population.

Cox proportional hazards regression models were used to estimate adjusted hazard ratios (HRs) of PC incidence associated with each exposure, stratified by age‐at‐risk (5‐year groups), and study area (10 areas), and adjusted for education (4 groups: no formal school, primary school, middle/high school, or college/university), body mass index (BMI), total physical activity, and, where appropriate, for smoking (3 groups: never, occasional, or ever regular), and alcohol (5 groups: abstainers, ex‐weekly drinkers, reduced‐intake drinkers, occasional drinkers, or weekly drinkers). The analysis for diet was also stratified by sex, and as a sensitivity analysis adjusted for other dietary variables. For analyses involving more than two categories, all HRs are presented with ‘floating’ standard errors to facilitate comparisons between any two groups 20. SAS version 9.3 and R version 2.14.2 were used for the analysis.

## Results

Among the 510,314 participants included, the mean (SD) baseline age was 51.5 (10.7) years, and 59% were women. Overall, 74% of men were ever‐regular smokers (including 6.7% who had stopped by choice), as opposed to only 3% of women. Among men, 33% drank at least weekly, much more than women did (2%), and the mean amount of alcohol consumed by weekly drinkers was 286 g/week for male (i.e. four drinks per day assuming one drink contains 10 g of pure alcohol) and 116 g/week (i.e. one to two drinks per day) for female drinkers. Among male weekly drinkers 37% reported having a heavy drinking episode (i.e. ≥60 g of alcohol in a drinking session) on a weekly basis.

During approximately 4.6 million person‐years of follow‐up, 688 participants developed PC between the ages 40–79 years, including 398 men and 290 women. Participants with PC were more likely to be older, male, from urban areas, regular smokers, and weekly drinkers, and to have higher mean SBP, random plasma glucose, and prevalent diabetes (Table 1). Standardised incidence rates in the 10 regions are shown in Table S1.

**Table 1:** Baseline characteristics of participants with or without incident pancreatic cancer

Variable^a^	Pancreatic cancer	
	No (*n* = 509,626)	Yes (*n* = 688)
Age (SD), year	51.5 (10.7)	60.7 (8.9)
Female, %	59.0	49.6
Urban residents, %	44.0	50.3
>6 years formal education, %	43.4	37.0
Annual household income ≥35,000 Yuan, %	24.7	28.7
Ever regular smoker, %		
Male	67.6	76.0
Female	2.8	2.3
Weekly drinker, %		
Male	33.3	45.8
Female	2.1	1.7
Alcohol weekly intake, g		
Male	285.6	330.4
Female	115.7	91.9
Heavy drinking^b^, %		
Male	37.2	42.8
Female	26.5	11.1
SBP (SD), mmHg	131.1 (21.3)	134.3 (21.7)
Random plasma glucose (SD), mmol/L	6.1 (2.3)	6.6 (3.5)
Total physical activity (SD), MET h/day	21.1 (13.9)	20.1 (14.2)
Overweight^c^, %	32.9	38.0
Obese^c^, %	4.1	6.5
BMI at baseline (SD), kg/m²	23.7 (3.4)	24.2 (3.6)
BMI at age 25 (SD), kg/m²	21.9 (2.6)	22.2 (2.8)
Waist circumference (SD), cm	80.3 (9.8)	82.0 (10.5)
Waist to hip ratio (SD)	0.88 (0.07)	0.89 (0.07)
Body fat percentage (SD), %	28.0 (8.4)	28.7 (9.2)
Height (SD), cm	158.7 (8.3)	158.3 (8.5)
Leg length (SD), cm	73.4 (4.8)	73.3 (4.8)
Self‐reported diabetes, %	3.1	6.5
Screen‐detected diabetes, %	2.7	6.3
Family history of diabetes, %	4.9	5.0
Family history of cancer, %	14.0	11.0

BMI, body mass index; SBP, systolic blood pressure; MET, metabolic equivalent of task.

a Results were adjusted for age, sex, and area (where appropriate).

b Heavy drinking was classified as the consumption of ≥60 g of alcohol on one occasion for men, and ≥40 g for women on a weekly basis, among weekly drinkers.

c Overweight: BMI ≥ 25 kg/m^2^; obese: BMI ≥ 30 kg/m^2^.

### Smoking, alcohol, and risk of PC among men

Compared with never smokers, current regular smokers had a 25% excess relative risk of PC (HR = 1.25, 95% CI 1.08–1.44), with a suggestive greater risk among former regular smokers (HR = 1.17, 0.82–1.65). The adjusted HR for current regular smokers appeared to be stronger among urban than rural men (HR = 1.46 [1.19–1.79] vs 1.04 [0.86–1.26]). Among current regular smokers, there was a nonsignificant dose–response relationship with amount smoked, with adjusted HRs of 1.19 (0.98–1.44), 1.25 (1.01–1.56), and 1.28 (0.93–1.74) for those who smoked <20, 20–24, and ≥25 cigarettes per day, respectively (*P* for trend = 0.28, Table 2). The risk of PC varied little by age of starting smoking regularly (*P* for trend = 0.30, Table 2).

**Table 2:** Adjusted HRs for PC by smoking status in male participants

	No. events	Rate per 100,000	HR (95% CI)^a^
Smoking category			
Never smokers	43	142.5	1.00 (0.73, 1.36)
Occasional smokers	29	123.2	1.16 (0.80, 1.67)
Former regular smokers	32	228.4	1.17 (0.82, 1.65)
Current regular smokers^b^	236	166.7	1.25 (1.08, 1.44)
Cigarette equivalents/day^c^			
Never smokers	43	142.5	1.00 (0.73, 1.38)
<20	109	157.2	1.19 (0.98, 1.44)
20–24	85	149.0	1.25 (1.01, 1.56)
≥25	42	143.8	1.28 (0.93, 1.74)
* P* for trend			*0.28*
Age of starting, years^c^			
Never smokers	43	142.5	1.00 (0.73, 1.38)
≥25	89	200.4	1.27 (1.03, 1.58)
20–24	82	140.9	1.18 (0.95, 1.47)
<20	65	122.7	1.20 (0.93, 1.54)
* P* for trend			*0.30*

a Models were stratified by age‐at‐risk and area, and adjusted for age at baseline, education, alcohol, BMI, and total physical activity.

b Regular smokers included former smokers who had stopped because of illness. Compared with never smokers, the adjusted HR was 1.28 (0.90–1.81) and 1.10 (0.75–1.62) for former smokers who had stopped due to illness and other reasons.

c Among current regular smokers.

Compared with abstainers, weekly drinkers had a 33% excess risk of PC (HR = 1.33, 1.11–1.58), with a suggestive greater risk among ex‐regular drinkers (HR = 1.32, 0.87–2.02), while men who drank occasionally, monthly, or reduced their alcohol intake were not at a higher risk (Table 3). Compared with abstainers, men who drank <140, 140–280, or 280–420 g/week had a nonsignificant 10% excess risk of PC (HR = 1.12 [0.81–1.55], 1.09 [0.78–1.53], and 1.14 [0.76–1.70], Table 3), whereas men who drank ≥420 g/week were at ~70% greater risk (HR = 1.69, 1.21–2.37). Moderate drinking (i.e. <280g/week) was not associated with risk of PC (HR = 1.09, 0.86–1.39), and similar associations were observed for men who drank spirits and beer or wine (HR = 1.05 [0.79–1.39] vs 1.09 [0.78–1.54]). Among weekly drinkers, heavy drinking on a weekly basis was associated with a 60% excess risk (HR = 1.60, 1.12–2.30), greater than the 43% excess risk associated with heavy drinking at special occasions (HR = 1.43, 0.90–2.28). Again, the excess risk associated with heavy drinking on a weekly basis was greater among urban than rural men (HR = 1.93 [1.29–2.87] and 1.35 [0.74–2.48] for ≥420 g/week). Moreover, the excess risk associated with heavy drinking appeared to be greater for spirits than for beer or wine (1.75 [0.91–3.36] vs 1.37 [0.45–4.16]). For both smoking and alcohol, additional adjustment for diabetes did not alter the associations (Table S2 and S3). Compared with men who were never smokers and abstainers, men who smoked ≥20 cigarettes per day and consumed ≥420 g of alcohol per week had an almost fourfold higher risk of PC (HR = 3.86, 1.65–8.97).

**Table 3:** Adjusted HRs for PC by alcohol drinking in male participants

Variable	No. events	Rate per 100,000	HR (95% CI)^a^
Drinking category			
Abstainers	72	169.5	1.00 (0.79, 1.27)
Occasional	81	123.0	1.04 (0.83, 1.30)
Monthly	11	83.8	0.95 (0.53, 1.73)
Reduced intake	18	174.9	1.06 (0.67, 1.69)
Ex‐regular	22	283.1	1.32 (0.87, 2.02)
Weekly	136	195.0	1.33 (1.11, 1.58)
Weekly intake (grams)			
Abstainers	72	169.5	1.00 (0.77, 1.29)
1–140	41	164.0	1.12 (0.81, 1.55)
140–280	34	180.1	1.09 (0.78, 1.53)
280–420	24	187.4	1.14 (0.76, 1.70)
≥420	37	283.5	1.69 (1.21, 2.37)
*P* for trend			*0.007*
Heavy drinking^b^			
No	78	178.2	Reference
Yes	58	444.4	1.60 (1.12, 2.30)

a Models were stratified by age‐at‐risk and area, and adjusted for age at baseline, education, smoking, BMI, and total physical activity.

b Heavy drinking was classified as the consumption of ≥60 g of alcohol on one occasion for men, and ≥40 g for women on a weekly basis, among weekly drinkers.

Among women, 290 developed PC between the ages 40–79 years during 2.4 million person‐years of follow‐up. Among the very few women who ever smoked regularly or drank weekly, there were 21 and 9 PC cases, respectively. Compared with never smokers, the adjusted HR for risk of PC was 1.00 (0.65–1.54) for current regular smokers, but the number of cases was too small to yield reliable estimates.

### Dietary factors and risk of PC

**Figure 1:**
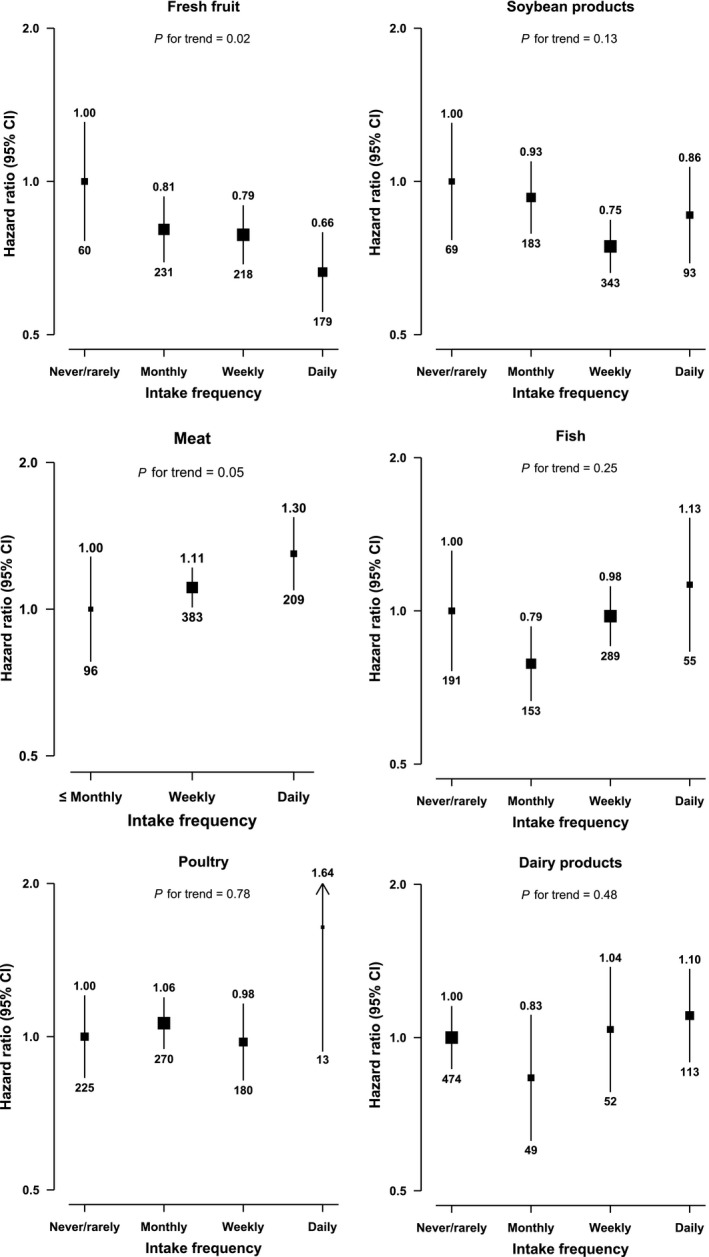
Adjusted HRs for PC by self‐reported intake frequency of dietary fraction. Intake frequency was classified as never/rarely (reference), monthly, weekly (1–3 days/week), and daily or almost daily (≥4 days/week and daily). The boxes represent hazard ratios and the vertical lines represent 95% confidence intervals. The sizes of the boxes are proportional to the inverse of the variance of the log hazard ratios. The numbers above the vertical lines are point estimates for hazard ratios, and the numbers below the lines are numbers of events. The models were stratified by age‐at‐risk, sex and study area, and adjusted for education, smoking, alcohol, BMI, and total physical activity.

Daily fresh fruit intake was associated with a lower risk of PC (Fig. 1). Compared with participants who reported consuming fresh fruit never or rarely, the adjusted HR was 0.66 (0.56–0.79) for those who consumed fresh fruit daily or almost daily. Consumption of red meat and fish were positively associated with risk of PC (Fig. 1). Compared with participants who ate red meat monthly or less frequently, the HR was 1.11 (0.98–1.19) and 1.30 (1.04–1.49) for those who ate red meat weekly and daily or almost daily, respectively. There was a suggestive inverse association between consumption of soybean products and risk of PC, with the adjusted HR comparing weekly with never or rarely being 0.75 (0.66–0.84). The association for each of these dietary factors did not change after further adjusting for consumption of other food or for diabetes (Table S4). There was no clear association of consumption of poultry, dairy products, or preserved vegetables with risk of PC (Fig. 1 and Table S4). As shown in Table S5, the associations of dietary factors with PC risk did not differ by sex (*P* for heterogeneity 0.25 for fruit and 0.79 for red meat, respectively).

## Discussion

This is the first large prospective study in China examining the associations of smoking, alcohol, and certain dietary factors with risk of PC. Among men, smoking and heavy alcohol consumption were associated with greater risk of PC. Moreover, low consumption of fresh fruit and high consumption of red meat were each associated with a higher risk of PC. Our results for smoking and alcohol are largely consistent with previous studies in Western and other East Asian populations. We further extended the results from previous studies by showing the relevance of fresh fruit and red meat consumption to risk of PC.

Several prospective studies from Western and other East Asian countries have previously examined the association of smoking with risk of PC 5, 21, 22. Although they tended to show reasonably consistently an excess risk among smokers, the strength of the association tended to vary between Western and East Asian populations and across different studies within specific populations. A meta‐analysis of 35 prospective cohort studies with 14,236 PC cases reported 70% and 20% excess risk among current and former smokers, respectively 5. Among current smokers, there were also moderate dose–response relationships with amount and duration smoked, with each 20 cigarettes per day and each 10 years smoking duration associated with 60% and 16% higher risk, respectively 5. Similar excess risks were also shown in a pooled analysis of 30 cohort studies from the Asia‐Pacific region that included 324 PC deaths 21. The observed HR for smoking in CKB was only about half as extreme as in previous studies of Western populations. In China, the proportion of adult men who have smoked cigarettes persistently since early adulthood is relatively low, especially in rural areas 23. Indeed, the HR for current smoking was less extreme in rural than urban areas in CKB (1.04 vs 1.46), but this urban versus rural difference may have been due to chance given the relatively small number of cases included.

Previous prospective studies have shown that heavy alcohol drinking (i.e. ≥3 drinks or 36 g alcohol per day) is associated with a 30% higher risk of PC, whereas the effects of light to moderate drinking remain unclear 6. However, previous evidence was primarily from European or North American populations and the excess risk for heavy alcohol drinking was inconsistent, with some studies showing a null result 6, 7, 8, 9. Studies conducted in Asian countries did not find an association of alcohol with risk of PC 24, 25, 26, possibly due to their small number of cases (mostly <200). Our study showed a strong association between heavy alcohol intake and PC risk (HR = 1.69 for ≥420 g/week), stronger than a recent meta‐analysis involving seven prospective cohort studies that included 2786 PC cases (HR = 1.17 for ≥5 drinks/day, equivalent to ≥420 g/week) 6. The stronger association in CKB might be related to the widespread use of distilled strong spirits (~80%)^14^ in China which might carry a higher risk than other types of alcohol, as shown in our study. In addition, it was hypothesized that the effects of alcohol on health in Asians may differ from those in Caucasians because many Asians cannot metabolize alcohol effectively due to presence of the so‐called Asian “flushing gene” 27, 28. On the other hand, our findings were consistent with previous studies in Western populations, showing that light or moderate drinking was not associated with risk of PC. In addition, our study showed that ex‐regular drinking was associated with a ~30% higher risk of PC, based on a small number of cases. This finding is not surprising given that the majority of ex‐regular drinkers stopped due to illness (~70%), suggesting that the positive association may be partly due to reverse causality bias.

Results from 11 case–control studies have consistently shown that high consumption of fruit (particularly citrus fruit) and vegetables are associated with a lower risk of PC, while high intake of red meat was associated with a higher risk 10. In contrast, several prospective cohort studies have showed null associations of intake of fruit, vegetable, red meat, or fish with risk of PC 10, 11, 12. However, meta‐analyses of those cohort studies yielded moderate to high heterogeneity between studies for fresh fruit (~50%) and red meat (~70%) that was unexplained 10, 11, 12. It is possible that previous studies assessed diet through different questionnaires and that diet patterns differed across populations, and therefore, making it challenging to combine their results in meta‐analysis. In addition, the majority of previous studies assessed a combination of fresh and processed fruit (e.g. frozen fruit, canned fruit, fruit juice), and provided limited data specifically on fresh fruit, which is often consumed raw as a snack in China. In our study, we found that high intake of fresh fruit was associated with a lower risk of PC, while the converse was true for intake of red meat and fish. Our risk estimate for red meat was consistent with a recent meta‐analysis of 16 prospective studies (8420 cases) that reported a pooled RR of 1.38 (1.05–1.81) comparing the top to the bottom category 29. A case–control study (451 cases and 1552 controls) in China reported an inverse association with fruit but a null association with red meat 30. However, this study was conducted two decades ago in an urban area and substantial changes in diet have taken place in China since then. Our study provided contemporary prospective evidence about the relevance of fresh fruit and red meat for PC risk in China, where consumption of fruit and red meat is relatively low, compared with Western countries. On the other hand, we found no associations of PC risk with several other dietary factors such as dairy products, poultry, and preserved vegetables.

Tobacco smoking and alcohol are classified as Group 1 carcinogens 31, 32. *N*‐nitrosamines are the major tobacco carcinogens 33, while acetaldehyde might be the most important carcinogen among ethanol metabolites 34. In addition, heavy alcohol intake may alter the inflammatory responses and increase production of reactive oxygen species, leading to DNA damage and dysregulation of cell proliferation and apoptosis 34, 35. Although the mechanism underlying the association of red meat with carcinogenesis is less clear, several hypotheses have been proposed. Red meat contains heme, and when cooked at high temperatures it produces heterocyclic amines (HCAs) and polycyclic aromatic hydrocarbons (PAHs) 36, which are considered probable carcinogens to humans 37, 38. On the other hand, fruit is a source of antioxidants, including carotenoids, flavonoids, and phenols 36. These anticarcinogenic agents can protect against cancer by trapping free radicals and reactive oxygen species 36, 39, possibly with complementary and overlapping influences on several pathways in carcinogenesis 39.

The strengths of the CKB include a prospective design, a large and diverse study population, ability to collect detailed information on smoking, alcohol, and diet, and careful adjustment for other risk factors for PC. This study also has limitations. First, we did not collect information on processed meat because its consumption is extremely low in China (<5 g/day) 15. Despite a possible carcinogenic role of HCAs and PAHs in processed meat 32, meta‐analysis of 14 prospective studies and 8564 PC cases showed a null association 36. Second, we were unable to properly examine the association of fresh vegetables with risk of PC because daily consumption is almost universal in our study population (95% daily intake). Third, dietary information at baseline was assessed with a simple qualitative food‐frequency questionnaire, and more detailed quantitative information on consumption was not collected 40. Fourth, we did not collect information about total energy intake. However, we adjusted the models for BMI and total physical activity which can be considered as a proxy of energy balance when examining the associations of diet and PC risk. Lastly, diet in our study was correlated with socioeconomic status, which is also associated with PC risk 4. Although we have adjusted for these factors, residual confounding by socioeconomic status may still exist.

In summary, smoking, heavy alcohol drinking (in men), low fruit consumption, and high red meat consumption were each associated with higher risk of developing PC in China. China now consumes approximately a third of the world's cigarettes, with a rapid increase since the 1980s 13. In China, annual alcohol per capita (aged 15 and above) alcohol consumption increased from less than 2 L in 1981 to almost about 7 L in 2010, with a projection of greater than 8 L by 2025 41, 42. Despite the increasing intake of fruit and stably high intake of vegetables in the past two decades, there has been a rapid increase in consumption of animal‐source foods, particularly red meat 15, 43. With these adverse trends in lifestyle and dietary factors, the risk of PC may increase further in China. These lifestyle risk factors are potentially modifiable, and our findings should inform development of disease prevention strategies for PC in the Chinese populations.

## Conflict of Interests

We declare that we have no conflict of interest.

## Supporting information


**Table S1.** Standardised incidence rate of PC by 10 regions.
**Table S2.** Adjusted HRs for PC by smoking status in male participants.
**Table S3.** Adjusted HRs for PC by alcohol in male participants.
**Table S4.** Adjusted HRs for PC by intake frequency of selected dietary fraction.
**Table S5.** Adjusted HRs for PC by intake frequency of selected dietary fraction.
