# Supplementary material for: Smoking, alcohol, and diet in relation to risk of pancreatic cancer in China: a prospective study of 0.5 million people
Source: Cancer Med. 2017 Dec 22;7(1):229–39. doi: 10.1002/cam4.1261 (PMC5773963; doi:10.1002/cam4.1261)
Supplement: Supplementary file 1 — Table S1. Standardised incidence rate of PC by 10 regions. Table S2. Adjusted HRs for PC by smoking status in male participants. Table S3. Adjusted HRs for PC by alcohol in male participants. Table S4. Adjusted HRs for PC by intake frequency of selected dietary fraction. Table S5. Adjusted HRs for PC by intake frequency of selected dietary fraction. [file CAM4-7-229-s001.docx]

**Supplementary Material**

**Smoking, alcohol and diet in relation to risk of pancreatic cancer in China: a prospective study of 0.5 million people**

**Cancer Medicine**

Running title: Smoking, alcohol, diet and pancreatic cancer

Yuanjie Pang^1^, ScM; Michael V Holmes^1,2^, MD, PhD; Yu Guo^3^, MSc; Ling Yang^1,2^, PhD; Zheng Bian^3^, MSc; Yiping Chen^1,2^, DPhil; Andri Iona^1,2^, MSc; Iona Y Millwood^1,2^, DPhil; Fiona Bragg^1^, DPhil; Junshi Chen^5^, MD; Liming Li^3,4^, MD, MPH; Christiana Kartsonaki^1,2^, DPhil; Zhengming Chen^1^, DPhil

1. Clinical Trial Service Unit & Epidemiological Studies Unit (CTSU), Nuffield Department of Population Health, University of Oxford, Oxford, UK
2. Medical Research Council Population Health Research Unit (MRC PHRU), Nuffield Department of Population Health, University of Oxford, Oxford, UK
3. Chinese Academy of Medical Sciences, 9 Dongdan San Tiao, Beijing 100730, China
4. School of Public Health, Peking University, Beijing 100191, China
5. National Center for Food Safety Risk Assessment, 37 Guangqu Road, Beijing 100021, China

**Address for correspondence**

Dr Christiana Kartsonaki

CTSU, Big Data Institute

Old Road Campus

University of Oxford

Oxford, OX3 7LF, UK

Fax: 44-1865-743985

Email: christiana.kartsonaki@ndph.ox.ac.uk

**Supplementary Table 1. Standardised incidence rate of PC by 10 regions**

| **Region** | **No. of events** | | **Standardised incidence rate^*^**  **(per 100,000 persons)** | |
| --- | --- | --- | --- | --- |
| **Urban** | | 353 | | 173.0 |
| Qingdao | | 58 | | 205.8 |
| Harbin | | 115 | | 204.0 |
| Haikou | | 14 | | 43.3 |
| Suzhou | | 122 | | 238.6 |
| Liuzhou | | 44 | | 87.3 |
| **Rural** | | 349 | | 136.1 |
| Sichuan | | 82 | | 171.7 |
| Gansu | | 41 | | 109.3 |
| Henan | | 51 | | 106.5 |
| Zhejiang | | 136 | | 246.0 |
| Hunan | | 39 | | 70.5 |

^*^ Results were standardised by age and sex.

**Supplementary Table 2. Adjusted HRs for PC by smoking status in male participants**

| **Variable** | **No. events** | **Rate**  **per 100,000** | **HR (95% CI)**^*^ |
| --- | --- | --- | --- |
| **Smoking category** |  |  |  |
| Never smokers | 43 | 142.5 | 1.00 (0.73, 1.36) |
| Occasional smokers | 29 | 123.2 | 1.16 (0.80, 1.67) |
| Former regular smokers | 32 | 228.4 | 1.17 (0.83, 1.66) |
| Current regular smokers^†^ | 236 | 166.7 | 1.25 (1.09, 1.44) |
|  |  |  |  |
| **Cigarette equivalents/day**^‡^ | |  |  |
| Never smokers | 43 | 142.5 | 1.00 (0.73, 1.38) |
| <20 | 109 | 157.2 | 1.19 (0.99, 1.45) |
| 20-24 | 85 | 149.0 | 1.25 (1.01, 1.56) |
| ≥25 | 42 | 143.8 | 1.27 (0.93, 1.73) |
| *p for trend* |  |  | *0.25* |
|  |  |  |  |
| **Age of starting, years**^‡^ |  |  |  |
| Never smokers | 43 | 142.5 | 1.00 (0.73, 1.38) |
| ≥25 | 89 | 200.4 | 1.28 (1.03, 1.58) |
| 20-24 | 82 | 140.9 | 1.19 (0.95, 1.47) |
| <20 | 65 | 122.7 | 1.20 (0.93, 1.54) |
| *p for trend* |  |  | *0.29* |

^*^ Models were stratified by age–at-risk and area, and adjusted for age at baseline, education, alcohol, BMI, total physical activity, and diabetes.

^†^ Regular smokers included former smokers who had stopped because of illness. Compared with never smokers, the adjusted HR were 1.27 (0.90, 1.80) and 1.11 (0.75, 1.62) for former smokers who had stopped due to illness, and due to other reasons, respectively.

^‡^ Among current regular smokers.

**Supplementary Table 3. Adjusted HRs for PC by alcohol in male participants**

| **Variable** | **No. events** | **Rate**  **per 100,000** | **HR (95% CI)**^*^ |
| --- | --- | --- | --- |
| **Drinking category** |  |  |  |
| Abstainers | 72 | 169.5 | 1.00 (0.79, 1.27) |
| Occasional | 81 | 123.0 | 1.05 (0.83, 1.31) |
| Monthly | 11 | 83.8 | 0.97 (0.53, 1.76) |
| Reduced intake | 18 | 174.9 | 1.05 (0.66, 1.66) |
| Ex-regular | 22 | 283.1 | 1.29 (0.85, 1.98) |
| Weekly | 136 | 195.0 | 1.34 (1.13, 1.60) |
|  |  |  |  |
| **Weekly intake** | |  |  |
| Abstainers | 72 | 169.5 | 1.00 (0.77, 1.29) |
| 0-140 g | 41 | 164.0 | 1.14 (0.83, 1.57) |
| 140-280 g | 34 | 180.1 | 1.12 (0.80, 1.56) |
| 280-420 g | 24 | 187.4 | 1.16 (0.77, 1.73) |
| ≥420 g | 37 | 283.5 | 1.71 (1.22, 2.39) |
| *p for trend* |  |  | *0.05* |
|  |  |  |  |
| **Heavy drinking**^†^ |  |  |  |
| No | 282 | 153.8 | Reference |
| Yes | 58 | 444.4 | 1.56 (1.09, 2.23) |

^*^ Models were stratified by age-at-risk and area, and adjusted for age at baseline, education, smoking, BMI, total physical activity, and diabetes.

^†^ Heavy drinking was classified as the consumption of ≥60 g of alcohol on one occasion for men, and ≥40 g for women on a weekly basis, among weekly drinkers.

**Supplementary Table 4. Adjusted HRs for PC by intake frequency of selected dietary fraction**

| **Variable** | **No. events** | **Model 1** | **Model 2** | **Model 3** |
| --- | --- | --- | --- | --- |
|  |  | **HR (95% CI)** | **(95% CI)** | **(95% CI)** |
| **Fresh fruit** |  |  |  |  |
| Never/rarely | 60 | 1.00 (0.77, 1.30) | 1.00 (0.77, 1.30) | 1.00 (0.77, 1.30) |
| Monthly | 231 | 0.81 (0.70, 0.93) | 0.81 (0.70, 0.93) | 0.85 (0.74, 0.99) |
| Weekly | 218 | 0.79 (0.69, 0.89) | 0.78 (0.68, 0.88) | 0.84 (0.74, 0.96) |
| Daily | 179 | 0.66 (0.56, 0.79) | 0.64 (0.53, 0.76) | 0.72 (0.61, 0.86) |
| *p for trend* |  | *0.02* | *0.009* | *0.05* |
|  |  |  |  |  |
| **Soybean products** | |  |  |  |
| Never/rarely | 69 | 1.00 (0.77, 1.29) | 1.00 (0.77, 1.30) | 1.00 (0.77, 1.29) |
| Monthly | 183 | 0.93 (0.79, 1.09) | 0.93 (0.80, 1.09) | 0.93 (0.79, 1.09) |
| Weekly | 343 | 0.75 (0.66, 0.84) | 0.74 (0.66, 0.84) | 0.74 (0.66, 0.83) |
| Daily | 93 | 0.86 (0.70, 1.06) | 0.86 (0.69, 1.06) | 0.84 (0.68, 1.04) |
| *p for trend* |  | *0.13* | *0.13* | *0.10* |
| **Meat** | |  |  |  |
| Never/rarely | 27 | 1.00 (0.66, 1.51) | 1.00 (0.66, 1.51) | 1.00 (0.66, 1.50) |
| Monthly | 69 | 0.99 (0.76, 1.28) | 1.00 (0.77, 1.30) | 0.99 (0.77, 1.29) |
| Weekly | 276 | 1.12 (1.01, 1.26) | 1.16 (1.04, 1.29) | 1.12 (1.01, 1.26) |
| Daily | 316 | 1.16 (1.01, 1.33) | 1.20 (1.04, 1.39) | 1.16 (1.01, 1.33) |
| *p for trend* |  | *0.32* | *0.24* | *0.33* |
| **Poultry** | |  |  |  |
| Never/rarely | 225 | 1.00 (0.84, 1.20) | 1.00 (0.82, 1.21) | 1.00 (0.84, 1.20) |
| Monthly | 270 | 1.06 (0.95, 1.19) | 1.05 (0.94, 1.16) | 1.06 (0.95, 1.18) |
| Weekly | 180 | 0.98 (0.83, 1.15) | 0.96 (0.80, 1.14) | 0.97 (0.82, 1.15) |
| Daily | 13 | 1.64 (0.95, 2.84) | 1.59 (0.91, 2.78) | 1.63 (0.94, 2.81) |
| *p for trend* |  | 0.78 | 0.91 | *0.82* |
|  |  |  |  |  |
| **Dairy products** | |  |  |  |
| Never/rarely | 474 | 1.00 (0.87, 1.15) | 1.00 (0.87, 1.15) | 1.00 (0.87, 1.15) |
| Monthly | 49 | 0.83 (0.63, 1.10) | 0.85 (0.64, 1.12) | 0.82 (0.62, 1.08) |
| Weekly | 52 | 1.04 (0.79, 1.37) | 1.07 (0.81, 1.41) | 1.02 (0.77, 1.34) |
| Daily | 113 | 1.10 (0.90,1.35) | 1.14 (0.93,1.40) | 1.06 (0.86, 1.30) |
| *p for trend* |  | *0.48* | *0.34* | *0.70* |
| **Fish** | |  |  |  |
| Never/rarely | 191 | 1.00 (0.77, 1.30) | 1.00 (0.76, 1.32) | 1.00 (0.77, 1.30) |
| Monthly | 153 | 0.79 (0.67, 0.93) | 0.79 (0.67, 0.93) | 0.79 (0.67, 0.92) |
| Weekly | 289 | 0.98 (0.86, 1.11) | 1.00 (0.88, 1.15) | 0.97 (0.85, 1.11) |
| Daily | 55 | 1.13 (0.84, 1.51) | 1.18 (0.88, 1.59) | 1.11 (0.83, 1.49) |
| *p for trend* |  | *0.25* | *0.16* | *0.26* |
| **Preserved vegetable** | |  |  |  |
| Never/rarely | 111 | 1.00 (0.82, 1.22) | 1.00 (0.82, 1.22) | 1.00 (0.82, 1.22) |
| Monthly | 199 | 1.11 (0.96, 1.30) | 1.13 (0.97, 1.31) | 1.12 (0.96, 1.30) |
| Weekly | 173 | 0.94 (0.81, 1.09) | 0.96 (0.83, 1.12) | 0.95 (0.81, 1.10) |
| Daily | 205 | 0.97 (0.82, 1.14) | 0.99 (0.84, 1.17) | 0.98 (0.83, 1.16) |
| *p for trend* |  | *0.44* | *0.59* | *0.51* |

^*^ Model1 was stratified by age-at-risk, sex, and area, and adjusted for age at baseline, education, smoking, alcohol, BMI, and total physical activity. Model 2 further adjusted for other food, and Model 3 further adjusted for diabetes.

**Supplementary Table 5. Adjusted HRs for PC by intake frequency of selected dietary fraction**

| **Variable** | **Male** | | **Female** | | ***P* for** |
| --- | --- | --- | --- | --- | --- |
|  | **No. events** | **HR (95% CI)** | **No. events** | **(95% CI)** | **heterogeneity** |
| **Fresh fruit** |  |  |  |  | *0.25* |
| Never/rarely | 35 | 1.00 (0.71, 1.41) | 25 | 1.00 (0.67, 1.50) |  |
| Monthly | 117 | 0.74 (0.61, 0.90) | 114 | 0.90 (0.73, 1.11) |  |
| Weekly | 100 | 0.68 (0.56, 0.82) | 118 | 0.91 (0.77, 1.08) |  |
| Daily | 88 | 0.76 (0.60, 0.97) | 91 | 0.59 (0.46, 0.76) |  |
| *p for trend* |  | *0.38* |  | *0.01* |  |
|  |  |  |  |  |  |
| **Soybean products** | |  |  |  | *0.44* |
| Never/rarely | 69 | 1.00 (0.77, 1.29) | 26 | 1.00 (0.66, 1.52) |  |
| Monthly | 183 | 0.93 (0.79, 1.09) | 76 | 0.90 (0.70, 1.14) |  |
| Weekly | 343 | 0.75 (0.66, 0.84) | 187 | 0.81 (0.70, 0.94) |  |
| Daily | 93 | 0.86 (0.70, 1.06) | 51 | 0.89 (0.67, 1.18) |  |
| *p for trend* |  | *0.61* |  | *0.10* |  |
|  |  |  |  |  |  |
| **Meat** |  |  |  |  | *0.79* |
| ≤Monthly | 35 | 1.00 (0.68, 1.48) | 61 | 1.00 (0.74, 1.35) |  |
| Weekly | 189 | 1.21 (1.07, 1.36) | 194 | 1.04 (0.91, 1.19) |  |
| Daily | 116 | 1.46 (1.16, 1.83) | 93 | 1.18 (0.93, 1.51) |  |
| *p for trend* |  | *0.40* |  | *0.32* |  |
|  |  |  |  |  |  |
| **Poultry** |  |  |  |  | *0.05* |
| Never/rarely | 80 | 1.00 (0.75, 1.34) | 145 | 1.00 (0.79, 1.26) |  |
| Monthly | 142 | 1.34 (1.15, 1.57) | 128 | 0.90 (0.77, 1.06) |  |
| Weekly | 108 | 1.25 (1.01, 1.55) | 72 | 0.80 (0.61, 1.04) |  |
| Daily | 10 | 2.51 (1.34, 4.69) | 3 | 0.88 (0.28, 2.76) |  |
| *p for trend* |  | *0.11* |  | *0.22* |  |
|  |  |  |  |  |  |
| **Dairy products** | |  |  |  | *0.24* |
| Never/rarely | 232 | 1.00 (0.82, 1.21) | 242 | 1.00 (0.82, 1.21) |  |
| Monthly | 25 | 0.92 (0.62, 1.35) | 24 | 0.76 (0.51, 1.12) |  |
| Weekly | 29 | 1.34 (.93, 1.93) | 23 | 0.80 (0.53, 1.20) |  |
| Daily | 54 | 1.23 (0.92, 1.66) | 59 | 0.99 (0.75, 1.32) |  |
| *p for trend* |  | *0.18* |  | *0.76* |  |
|  |  |  |  |  |  |
| **Fish** |  |  |  |  | *0.09* |
| Never/rarely | 69 | 1.00 (0.64, 1.55) | 122 | 1.00 (0.72, 1.39) |  |
| Monthly | 80 | 1.09 (0.87, 1.38) | 73 | 0.62 (0.50, 0.78) |  |
| Weekly | 155 | 1.31 (1.11, 1.56) | 134 | 0.80 (0.66, 0.97) |  |
| Daily | 36 | 1.69 (1.17, 2.43) | 19 | 0.77 (0.47, 1.27) |  |
| *p for trend* |  | *0.05* |  | *0.69* |  |
|  |  |  |  |  |  |
| **Preserved vegetable** | |  |  |  | *0.70* |
| Never/rarely | 56 | 1.00 (0.75, 1.33) | 55 | 1.00 (0.76, 1.32) |  |
| Monthly | 93 | 0.92 (0.74, 1.15) | 106 | 1.33 (1.08, 1.63) |  |
| Weekly | 84 | 0.83 (0.67, 1.03) | 89 | 1.05 (0.85, 1.29) |  |
| Daily | 107 | 0.96 (0.77, 1.21) | 98 | 0.97 (0.77, 1.22) |  |
| *p for trend* |  | *0.80* |  | *0.41* |  |

^*^ Estimates were stratified by age-at-risk and area, and adjusted for age at baseline, education, smoking, alcohol, BMI, and total physical activity.
